# Supplementary material for: Endogenous opioids regulate moment-to-moment neuronal communication and excitability
Source: Nat Commun. 2017 Mar 22;8:14611. doi: 10.1038/ncomms14611 (PMC5364458; doi:10.1038/ncomms14611)
Supplement: Supplementary Information — Supplementary Figure. [file ncomms14611-s1.pdf]

SUPPLEMENTARY INFORMATION

Supplementary Figure 1.

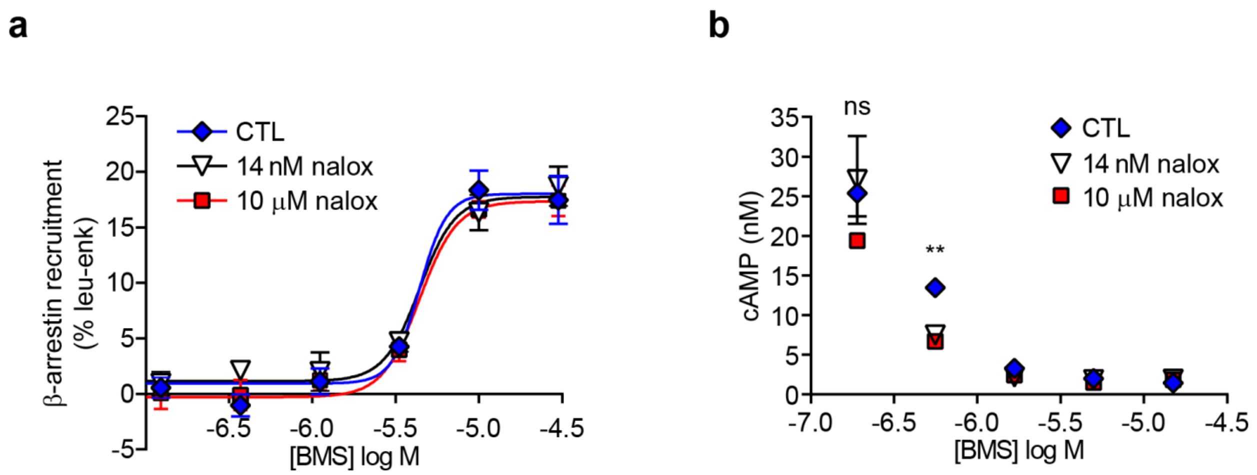

Supplementary Figure 1. Agonist activity of BMS-198187 is not blocked by the orthosteric antagonist naloxone. Assays of β-arrestin recruitment or cAMP accumulation were used on CHO-OPRD1 cells, stably transfected with DOR to assess BMS-198187 (BMS) agonist activity in the presence/absence of naloxone at control (CTL, no naloxone), low (14 nM) and high (10 μM) concentrations. (**a**) dose-response curves (n=6) showing BMS-198187 increased β-arrestin recruitment in a dose dependent manner under control conditions (blue, diamond) which was unaffected by low (white, triangle) or high (red, square) concentrations of naloxone. (**b**) CHO-ORPD1 cells were stimulated with 5 μM forskorlin in the presence/absence of differing concentrations of BMS-198187 and naloxone (n=4). In the absence of BMS-198187, naloxone (10 μM) had no effect on total cAMP levels when compared to control forskorlin stimulation (CTL: 29.7 ± 1.8 nM, n=4, grey line; naloxone: 30.6 ± 3.5 nM, n=4, black line; $p<0.05$, unpaired *t*-test). BMS-198187 completely abolished cAMP detection at concentrations higher than 1.67 μM, indicating full agonist activity at DOR. This was unaffected by naloxone at both low and high concentrations. At lower concentrations of BMS-198187 (< 560 nM), naloxone seemed to reduced cAMP levels which is opposite to the expected outcome of an opioid receptor antagonist, which would ordinarily increase cAMP levels by inhibiting $G\alpha_{i/o}$ signaling. These data provide strong evidence to indicate BMS-198187 agonist activity is not inhibited by the orthosteric ligand naloxone. Thus any action of BMS-198187 that is reversed by naloxone is likely to be through PAM activity of BMS-198187 which is acting with and enhancing the signaling of an orthosteric agonist. Data are represented as mean ± S.E.M.
